# Therapeutic Targeting of Eosinophil Adhesion and Accumulation in Allergic Conjunctivitis

**DOI:** 10.3389/fphar.2012.00203

**Published:** 2012-12-26

**Authors:** Monica Baiula, Andrea Bedini, Gioia Carbonari, Samantha Deianira Dattoli, Santi Spampinato

**Affiliations:** ^1^Department of Pharmacy and Biotechnology, University of BolognaBologna, Italy

**Keywords:** eosinophil, inflammation, allergic conjunctivitis, adhesion molecules, antihistamine, glucocorticoid

## Abstract

Considerable evidence indicates that eosinophils are important effectors of ocular allergy. Increased worldwide prevalence of allergic eye pathologies has stimulated the identification of novel drug targets, including eosinophils and adhesion molecules. Accumulation of eosinophils in the eye is a key event in the onset and maintenance of allergic inflammation and is mediated by different adhesion molecules. Antihistamines with multiple mechanisms of action can be effective during the early and late phases of allergic conjunctivitis by blocking the interaction between β_1_ integrins and vascular cell adhesion molecule (VCAM)-1. Small molecule antagonists that target key elements in the process of eosinophil recruitment have been identified and reinforce the validity of α_4_β_1_ integrin as a therapeutic target. Glucocorticoids are among the most effective drugs for ocular allergy, but their use is limited by adverse effects. Novel dissociated glucocorticoids can prevent eosinophil accumulation and induce apoptosis of eosinophils, making them promising candidates for ophthalmic drugs. This article reviews recent understanding of the role of adhesion molecules in eosinophil recruitment in the inflamed conjunctiva along with effective treatments for allergic conjunctivitis.

## Introduction

Ocular allergy is a disease primarily characterized by an inflammatory response of the conjunctival mucosa (Origlieri and Bielory, 2009). Allergic conjunctivitis is the most common form of ocular allergy. The term “allergic conjunctivitis” refers to a collection of disorders that affect the lid, conjunctiva, and/or cornea. Although allergic conjunctivitis is not generally life threatening, the symptoms of ocular allergy may have a significant impact on quality of life. Ocular symptoms are one of the most frequent reasons for consultation among patients with allergic rhinoconjunctivitis (Abelson et al., 2003). Acute forms of ocular allergy involve transient symptoms of itching, tearing, and swelling, while chronic allergies are sight-threatening and exhibit symptoms such as severe pain and visual disturbances (Leonardi, 1999).

Ophthalmic anti-allergic treatment includes topical mast cell stabilizers, oral and topical antihistamines, antihistamine-vasoconstrictor combinations, dual action agents with mast cell-stabilizing, and antihistaminic properties, and anti-inflammatory agents including steroids and non-steroidal drugs. Topical ocular corticoids are very effective, but display frequent side effects such as glaucoma, cataracts, and corneal ulcers (Ono and Abelson, 2005).

In the following sections, we review eosinophil adhesion molecules and the mechanisms involved in the recruitment of eosinophils to the allergic conjunctiva. Cell adhesion-based therapeutic strategies and the use of dissociated glucocorticoid in allergic conjunctivitis are also conceptualized.

## Contribution of Eosinophils to Conjunctival Inflammation

The ocular allergic response results from exposure of the conjunctiva to an environmental allergen with subsequent binding of specific immunoglobulin E molecules onto conjunctival mast cells. This immediate response lasts for 20–30 min and induces enhanced tear levels of histamine, tryptase, prostaglandins, and leukotrienes. Mast cell degranulation also induces activation of vascular endothelial cells and expression of chemokines and adhesion molecules (Ono and Abelson, 2005). These factors initiate the recruitment phase of inflammatory cells into the conjunctival mucosa, which leads to the late phase reaction. The late phase corresponds to the persistent clinical inflammation observed in perennial and chronic allergic diseases (Bacon et al., 2000), and is characterized by the mucosal infiltration of eosinophils, neutrophils, basophils, and T lymphocytes. In addition, conjunctival and corneal epithelial cells and fibroblasts may contribute to the allergic inflammatory response by inducing expression of cytokines, chemokines, adhesion molecules, and factors that maintain local inflammation and lead to tissue remodeling (Kumagai et al., 2006; Leonardi et al., 2006).

Eosinophils are multifunctional leukocytes implicated in the pathogenesis of numerous inflammatory processes including parasitic helminth infections and allergic diseases (Rothenberg, 1998). In response to diverse stimuli, eosinophils are recruited from the circulation into inflammatory foci, where they modulate immune responses through an array of mechanisms. Eosinophils are produced from pluripotent stem cells in bone marrow, and their migration into the circulation is primarily regulated by interleukin (IL)-5 (Collins et al., 1995). Circulating eosinophils are subsequently extravasated by a regulated process involving the coordinated interaction between chemokines, adhesion molecules (α_4_β_1_, α_4_β_7_, α_M_β_2_, α_L_β_2_ integrins), and adhesion receptors on the endothelium including mucosal addressin cell adhesion molecule (MAdCAM)-1, vascular cell adhesion molecule (VCAM)-1, and intercellular adhesion molecule (ICAM)-1.

Activation of eosinophils by cytokines, immunoglobulins, and complement can lead to the secretion of an array of chemokines and lipids (Kita, 1996), which have proinflammatory effects including upregulation of adhesion systems, modulation of cellular trafficking, and activation and regulation of vascular permeability, mucus secretion, and smooth muscle constriction. Therefore, it is reasonable to consider eosinophils to be multifaceted leukocytes that contribute to various physiological and pathological processes depending on their location and activation status.

## Role of Adhesion Molecules in the Multistep Paradigm of Eosinophil Trafficking during Inflammation

**Figure 1:**
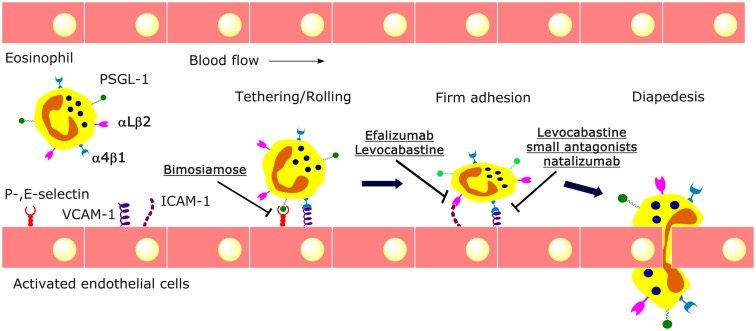
**Multistep process of eosinophil trafficking**. Circulating leukocytes initially tether and roll on endothelium via selectins and α_4_β_1_ integrin, followed by firm adhesion to endothelium via β_1_ and β_2_ integrins, and subsequent extravasation between endothelial cells. Cell adhesion-based therapeutic strategies specifically target adhesion molecules. For example, bimosiamose, a selectin antagonist, blocks the recruitment of eosinophils mediated by E-selectin. The antihistamine levocabastine, several small antagonists, and the monoclonal antibody natalizumab can interfere with the interaction between α_4_β_1_ integrin and VCAM-1, while efalizumab and levocabastine can affect α_L_β_2_/ICAM-mediated adhesion.

Accumulating evidence indicates that the selective recruitment of eosinophils is elegantly orchestrated by interactions between chemokines and adhesion molecules (Ebnet et al., 1996). Such translocations to tissue sites prime eosinophils to become even more susceptible to subsequent stimuli (so-called outside-in signaling; Bochner, 2000). This mechanism not only facilitates eosinophil functions within the tissue but may also prevent systemic over-activation of circulating eosinophils. The recruitment of eosinophils to inflamed tissue is a multistage process in which eosinophils undergo (1) priming, (2) rolling and tethering along endothelial cells, (3) firm adhesion to the endothelium, (4) transendothelial diapedesis, and (5) chemotaxis to the inflammatory site (Broide and Sriramarao, 2001; Figure 1).

Eosinophils express numerous adhesion molecules. Most attention has focused on the highly expressed integrins including α_4_β_7_, the CD18 family of molecules (β_2_-integrins), and the very late antigen (VLA)-4 molecules (β_1_ integrins; Bochner and Schleimer, 1994). The CD18 family of molecules includes lymphocyte function antigen (LFA)-1 and Mac-1, which interact with endothelial cells via ICAM-1. VLA-4 interacts with endothelium via VCAM-1 or fibronectin (FN), while α_4_β_7_ integrin interacts with MAdCAM-1 on vascular endothelium. These integrins have variable roles in eosinophil trafficking during inflammation. It is now clear that engagement of eosinophil adhesion molecules with their ligands not only induces a pro-adhesive pathway, but also activates expression of proinflammatory genes that propagate eosinophil survival by a paracrine pathway.

The first step of eosinophil recruitment involves tethering to the endothelium mediated by P-selectin glycoprotein ligand-1, l-selectin, and P-selectin, followed by α_4_ integrin-dependent rolling along the vessel wall. Following activation by selective chemokines (eotaxin and CCL5/RANTES), rolling eosinophils engage α_4_ (α_4_β_1_ and α_4_β_7_) and β_2_ (α_M_β_2_ and α_L_β_2_) integrins to firmly adhere to vascular endothelial cells. This process requires firm adhesion of eosinophils mediated by α_4_β_1_ integrin-VCAM-1 interactions, increased affinity of β_2_ integrins for ICAM-1, and then detachment of α_4_β_1_ integrin from VCAM-1 (Kuijpers et al., 1993; Alblas et al., 2001). If detachment of α_4_β_1_ integrin from VCAM-1 is inhibited, eosinophils will remain stuck to the endothelium, and will not undergo transendothelial migration (Kuijpers et al., 1993). In contrast to the predominant role for VCAM-1 in the recruitment of eosinophils, recruitment of lymphocytes is far less dependent on VCAM-1 (Barthel et al., 2006). Finally, in the presence of chemotactic gradients, adherent eosinophils migrate between endothelial cells to facilitate subsequent extravasation or migration of eosinophils into tissues (Edwards et al., 2000).

The identification of molecules that specifically regulate eosinophil function and/or production offers a new therapeutic strategy that may be useful for the treatment of allergic and inflammatory pathologies. Agents that interrupt eosinophil adhesion to the endothelium by disrupting interactions between CD18 and ICAM-1 or VLA-4 and VCAM-1 are currently being developed.

## Cell Adhesion-Based Therapeutic Strategies

Adhesion molecules and their intracellular signals are targets for intervention to disrupt eosinophil recruitment. In recent years, advancement in understanding of the pathophysiology of ocular allergies has paved the way for the development of newer drug candidates. Inhibitors of selectins and integrins have been used to block eosinophilia and inflammation in research studies and clinical trials. The small molecule pan-selectin antagonist bimosiamose (TBC-1269) blocks E-selectin-mediated leukocyte recruitment and attenuates late asthmatic reactions after allergen challenge in mild asthmatics (Romano, 2005; Beeh et al., 2006; Figure 1).

Several small molecule antagonists of α_4_β_1_ integrin have been developed. Compound A, a non-peptidyl small molecule, blocks eosinophil accumulation in the lungs of mice that have been challenged with ovalbumin (Koo et al., 2003). WAY103 blocks the binding of human eosinophils to recombinant human VCAM-1 (Sedgwick et al., 2005). TR14035, an orally active dual antagonist of the α_4_β_1_/α_4_β_7_ integrins, reduces eosinophil infiltration into the rat lung by inhibiting leukocyte rolling and adhesion (Cortijo et al., 2006). Valategrast (R411), a small molecule antagonist of α_4_β_1_ integrin, represents a potential therapy for asthma (Woodside and Vanderslice, 2008).

Efalizumab (Raptiva) is a monoclonal antibody directed against CD11a, the alpha subunit of LFA-1. Efalizumab inhibits LFA-1 (Li et al., 2009) by sterically hindering the interaction between LFA-1 and ICAM-1 (Krueger, 2002; Jullien et al., 2004). Efalizumab also inhibits the extravasation and activation of T lymphocytes, as well as interactions between T lymphocytes and keratinocytes (Jullien et al., 2004; Schön, 2008). Efalizumab was approved for the treatment of moderate to severe plaque psoriasis (Frampton and Plosker, 2009), but was withdrawn from the market in 2009 because of three cases of progressive multifocal leukoencephalopathy (Korman et al., 2009).

The validity of VLA-4 as a therapeutic target in humans has been confirmed by natalizumab (Tysabri^™^), which was the first adhesion molecule antagonist tested in a clinical trial for patients with multiple sclerosis and other inflammatory disorders (Miller et al., 2003). Natalizumab is a humanized recombinant monoclonal antibody that binds to the alpha subunits of the α_4_β_1_ and α_4_β_7_ integrins. Natalizumab reduces extravasation of leukocytes into peripheral tissues by interfering with the physical interaction of α_4_β_1_ integrin with its natural ligands, VCAM-1 and FN (Stüve and Bennett, 2007). Nevertheless, the use of natalizumab is complicated by rare cases of progressive multifocal leukoencephalopathy (Kleinschmidt-Demasters and Tyler, 2005; Langer-Gould et al., 2005).

The adhesion-based therapeutic strategies targeting α_4_ integrin seem promising for ocular allergy. In addition to its effects on H_1_ histamine receptors, levocabastine, but not the first-generation antihistamine chlorpheniramine, binds to human integrin α_4_β_1_ and prevents eosinophil adhesion to VCAM-1, FN, and human umbilical vascular endothelial cells *in vitro*. Similarly, levocabastine affects α_L_β_2_/ICAM-1-mediated adhesion of Jurkat cells. In a model of allergic conjunctivitis, levocabastine eye drops reduced the clinical aspects of the late phase reaction and the conjunctival expression of α_4_β_1_ integrin by reducing eosinophil infiltration (Qasem et al., 2008). These data confirm that levocabastine is an antihistamine with multiple mechanisms of action that inhibit the early and late phases of allergic reaction (Bielory et al., 2005). Moreover, topical levocabastine reduces the expression of ICAM-1 on epithelial conjunctival cells *in vivo* and *in vitro* (Buscaglia et al., 1996; Bielory et al., 2005). Other anti-allergic agents, such as azelastine (Ciprandi et al., 1996), cetirizine, olopatadine (Schultz, 2006), or fexofenadine (Ciprandi et al., 2003) may also reduce ICAM-1 expression. In contrast, the antihistamine levocetirizine inhibits adhesion of eosinophils to VCAM-1 (Wu et al., 2005). In patients with allergic conjunctivitis, H_1_-antihistamines relieve more relevant symptoms, such as itching, erythema, tearing, and edema, and have a more favorable benefit-risk ratio than all other classes of medications (Anonymous, 2010; Bohets et al., 2011).

Use of antihistamines in allergic conjunctivitis has evolved, and knowledge of the mechanisms of disease has progressed. Histamine is more specifically antagonized by second generation antihistamines, while newly formed mediators and downstream effectors (prostaglandins, leukotrienes, ILs, adhesion molecule, tumor necrosis factor, eosinophils, and neutrophils) are more selectively antagonized by dual/multiple-action agents, such as ketotifen, olopatadine, bepotastine, and alcaftadine, which have been shown to have effects on the early and late phases of ocular allergy. In fact, these drugs act as H1 receptor antagonists and mast cell stabilizers. Moreover, both ketotifen and olopatadine were able to decrease the expression of inflammatory markers and adhesion molecules on conjunctival cells in patients with allergic conjunctivitis (Avunduk et al., 2005). Some of the third generation antihistamines inhibit the vacuolization and accumulation of eosinophils after allergen challenge and directly inhibit eosinophils *in vitro* (Rothenberg and Hogan, 2006). Bepotastine, an antihistamine with mast cell-stabilizing activity, also has been shown to inhibit the late phase reaction through multiple mechanisms including histamine H_1_ receptor antagonism, mast cell stabilization, and inhibition of eosinophil migration to ocular inflammatory sites (Kida et al., 2010). Rupatadine is a selective and long-acting new drug with a strong antagonistic activity toward both histamine H1 receptors and platelet-activating factor receptors. It showed potent anti-allergic activity *in vitro*, including inhibition of mast cell degranulation and eosinophil chemotaxis, and *in vivo* (Sudhakara et al., 2009); moreover, rupatadine can inhibit CD18 and CD11b (Barron et al., 2004).

## Glucocorticoids Modulate Eosinophil Accumulation in Allergic Conjunctivitis

Glucocorticoids are potent drugs and among the most effective for the treatment of allergic eye disease (Ono and Abelson, 2005). Their efficacy is due in part to the prevention of eosinophil accumulation, activation, and induction of eosinophil apoptosis, suppression of the synthesis and release of eosinophil survival factors, and stimulation of eosinophil engulfment by phagocytic cells (Druilhe et al., 2003). Glucocorticoids like hydrocortisone, triamcinolone, fluromethalone, rimexalone, prednisolone, and dexamethasone have been widely used in the treatment of allergic conjunctivitis (Mishra et al., 2011).

Glucocorticoids reduce eosinophilia by suppressing transcription of a number of genes for inflammatory mediators including IL-3, IL-4, IL-5, granulocyte macrophage colony-stimulating factor, and various chemokines including the eotaxins. One of the main actions of glucocorticoids on eosinophil-activating cytokines is destabilization of mRNA, resulting in reduced half-life of cytokines such as eotaxins (Stellato et al., 1999). In addition, glucocorticoids inhibit the cytokine-dependent survival of eosinophils (Schleimer and Bochner, 1994).

Concerning glucocorticoid treatment of the eyes, the therapeutic effects are powerful and rapid, but, unfortunately, their anti-inflammatory and immunosuppressive effects frequently occur with significant adverse effects that may limit their use (Frauman, 1996). At the ocular level, classical glucocorticoids may cause elevation of intraocular pressure, which may lead to glaucoma (Kersey and Broadway, 2006) and cataract formation (Carnahan and Goldstein, 2000). There is, therefore, a pressing need for compounds with the anti-inflammatory potency of standard glucocorticoids but fewer undesired side effects.

Glucocorticoids exert their actions as a consequence of penetrating the cytoplasm and binding to the glucocorticoid receptor. The glucocorticoid–glucocorticoid receptor complex reaches the nucleus and acts as a transcription factor by binding to specific DNA sites and modifying transcription. This can have two effects on gene transcription. It can either activate transcription (transactivation) by directly binding to the promoter region of target genes or by interacting with other transcription factors, such as activator protein-1, nuclear factor-kappa B, and others, or it can suppress transcription (transrepression; Biddie and Hager, 2009).

It has been hypothesized that transrepression is the key mechanism of the anti-inflammatory effects of glucocorticoids, whereas transactivation has been assumed to cause side effects. In fact, transactivation activity has been implicated in many of the adverse effects associated with glucocorticoid therapy (Schäcke and Rehwinkel, 2004); the glucocorticoid-induced elevation of intraocular pressure seems to be primarily related to transactivation. Although glucocorticoids enhance the transactivation and expression of anti-inflammatory genes including lipocortin-1 and inhibitor of nuclear factor-kappa B, the transrepression mechanism may not be sufficient to explain their anti-inflammatory effects (Reichardt et al., 2001). However, in an admittedly oversimplified model, the dissociation of transrepression and transactivation has long been considered to be pivotal to the separation of the therapeutic and side effects of glucocorticoid therapy. Many researchers are trying to develop new drugs, dissociated compounds, or selective glucocorticoid receptor agonists (SEGRAs) that should preserve the beneficial anti-inflammatory activity but offer a better side effect profile. The identification of SEGRAs with separable transrepression and transactivation activities represents an important research goal in steroid pharmacology.

Mapracorat (also known as ZK245186 or BOL-303242-X) is a novel selective glucocorticoid receptor agonist that maintains a beneficial anti-inflammatory activity but seems to be less effective in transactivation, resulting in a lower potential for side effects. Mapracorat has been proposed for topical treatment of inflammatory skin and ocular disorders. In cultured human eosinophils, mapracorat shows the same potency as dexamethasone but displays higher efficacy in increasing spontaneous apoptosis and counteracting cytokine-sustained eosinophil survival (Baiula et al., 2011). In an *in vivo* model of allergic conjunctivitis, mapracorat, or dexamethasone eye drops induce an analogous reduction in clinical symptoms and conjunctival eosinophil accumulation (Baiula et al., 2011).

Recently, it was reported that mapracorat acts as a partial glucocorticoid receptor agonist at therapeutic doses and elicits a lower myocilin expression profile in monkey trabecular meshwork cells in comparison with traditional ocular steroids (Cavet et al., 2010). Mapracorat that is topically administered as eye drops displays a reduced ability to increase intraocular pressure in normotensive rabbits when compared to dexamethasone (Shafiee et al., 2011).

Mapracorat appears to be a promising candidate for topical treatment of allergic eye disorders and is under investigation in several clinical trials. Mapracorat maintains an anti-allergic profile similar to that of dexamethasone, but seems to have fewer transactivation effects in comparison to that classical glucocorticoid. The inhibitory effect of mapracorat on eosinophil accumulation observed *in vivo* at the conjunctival level may involve various mechanisms including eosinophil apoptosis and recruitment and activation or release of cytokines and chemokines. The contribution of glucocorticoids to eosinophil apoptosis in allergic diseases *in vivo* remains to be investigated further.

Several pharmaceutical companies have been trying to discover SEGRAs with therapeutically beneficial profiles (Berlin, 2010). Intensive research efforts led to the discovery of promising compounds that are at the preclinical developmental stage or early clinical stage. It is uncertain whether a complete separation of transactivation and transrepression will result in compounds with decreased adverse effect profiles. Although transactivation is thought to be responsible for the deleterious side effects of chronic glucocorticoid treatment, some transactivation events have shown therapeutic benefit. Indeed, the anti-inflammatory process is mediated by transactivating, transrepressing, and non-genomic actions of the glucocorticoid receptor (Rhen and Cidlowski, 2005), suggesting that the search for truly dissociated drugs in the treatment of inflammation may not be necessary. Rather, it may be necessary to search for “differential” glucocorticoid receptor agonists that show the most favorable functional profiles.

## Conclusion

Several drug targets in allergic conjunctivitis have been identified in the past several years. These advancements have opened the way for the development of newer drug candidates and for a better understanding of the mechanisms of action of currently used drugs.

Based on available research, dual action antihistamines are efficacious for the treatment of allergic conjunctivitis. Multiple mechanisms of action exist for antihistamines such as levocabastine. Targeting adhesion molecules appears to be particularly interesting. This contributes to improved effects on early (mediated by antihistamine action) and late (mediated by blocking integrin-mediated cell adhesion) phases of ocular allergy.

Glucocorticoids are among the most effective anti-inflammatory drugs employed for allergic eye diseases, although the risk of adverse effects may limit their use. Glucocorticoids that dissociate transrepression and transactivation are actively being pursued in order to better separate therapeutic and side effects. Mapracorat, a promising dissociated SEGRA candidate for the treatment of allergic conjunctivitis, can prevent eosinophil accumulation and activation and induce eosinophil apoptosis *in vitro*. These effects have been confirmed *in vivo* using a model of allergic conjunctivitis. In fact, mapracorat was able to increase apoptosis of eosinophils in the conjunctival tissue of ovalbumin-sensitized guinea pigs (Baiula et al., unpublished results).

Improved comprehension of the molecular mechanisms of action of drugs used in ocular allergy will be valuable for increasing the efficacy and safety of drugs and for the discovery of novel therapies.

## Conflict of Interest Statement

The authors declare that the research was conducted in the absence of any commercial or financial relationships that could be construed as a potential conflict of interest.
